# BDNF–TrkB signaling in the nucleus accumbens shell of mice has key role in methamphetamine withdrawal symptoms

**DOI:** 10.1038/tp.2015.157

**Published:** 2015-10-27

**Authors:** Q Ren, M Ma, C Yang, J-C Zhang, W Yao, K Hashimoto

**Affiliations:** 1 Division of Clinical Neuroscience, Chiba University Center for Forensic Mental Health, Chiba, Japan

## Abstract

Depression is a core symptom of methamphetamine (METH) withdrawal during the first several weeks of abstinence. However, the precise mechanisms underlying METH withdrawal symptoms remain unknown. Brain-derived neurotrophic factor (BDNF) and its specific receptor, tropomyosin-related kinase (TrkB), have a role the in pathophysiology of depression. In this study, we examined the role of BDNF–TrkB signaling in different brain regions of male mice with METH withdrawal symptoms. Repeated METH (3 mg kg^−1^ per day for 5 days) administration to mice caused a long-lasting depression-like behavior including anhedonia. Western blot analysis showed that BDNF levels in the nucleus accumbens (NAc) of METH-treated mice were significantly higher than those of control mice whereas BDNF levels in other regions, including the prefrontal cortex and hippocampus, were not altered. METH-induced depression-like behavior, behavioral sensitization and dendritic changes in the NAc shell were improved by subsequent subchronic administration of TrkB antagonist ANA-12 (0.5 mg kg^−1^ per day for 14 days), but not TrkB agonist 7,8-dihydroxyflavone (10 mg kg^−1^ per day for 14 days). *In vivo* microdialysis showed that METH (1 mg kg^−1^)-induced dopamine release in NAc shell of METH-treated mice was attenuated after subsequent subchronic ANA-12 administration. Interestingly, a single bilateral infusion of ANA-12 into the NAc shell, but not NAc core, showed a rapid and long-lasting therapeutic effect. However, ketamine and paroxetine had no effect. These findings suggest that increased BDNF–TrkB signaling in the NAc shell has an important role in the behavioral abnormalities after withdrawal from repeated METH administration, and that TrkB antagonists are potential therapeutic drugs for withdrawal symptoms in METH abusers.

## Introduction

Abuse of methamphetamine (METH) is a major public health problem. METH is a powerfully addictive stimulant associated with serious health risks such as cognitive impairment, aggression, psychotic symptoms and behavior, and potential heart and brain damage.^1, 2, 3, 4, 5, 6^ In humans, many psychiatric and psychological symptoms emerge after withdrawal from the repeated use of METH. Major METH withdrawal symptoms include depression (for example, anhedonia, dysphoria and anergia), agitation and irritability, fatigue (for example, increased sleeping and inactivity) and cognitive impairment. These symptoms may last from a few days to a few months.^7^ Withdrawal symptoms have been linked to a propensity for METH abuse relapse. METH-dependent subjects exhibit a wide range in the severity of depressive symptoms with psychotic symptoms.^8^ Thus, depression is a core symptom of METH withdrawal during the first several weeks of abstinence.^7, 8, 9, 10^ Although antidepressants are used for treating these symptoms, their efficacy is limited. To date, the precise mechanisms underlying METH withdrawal symptoms remain unknown.

Accumulating evidence suggests that the brain-derived neurotrophic factor (BDNF) and its specific receptor, tropomyosin-related kinase (TrkB) signaling, have an important role in the pathophysiology of depression and drug addiction.^11, 12, 13, 14, 15, 16, 17, 18, 19, 20, 21, 22^ This would make BDNF–TrkB signaling a potential therapeutic target for depression and drug addiction. Previously, we reported that pretreatment with 7,8-dihydroxyflavone (7,8-DHF), a TrkB agonist, is able to attenuate behavioral abnormalities (for example, hyperlocomotion, prepulse inhibition deficits, development of behavioral sensitization) after METH administration.^23, 24^ Furthermore, infusion of antibodies against BDNF or TrkB into the nucleus accumbens (NAc) attenuates the stimulation of dopamine release and behavioral abnormalities after METH exposure.^25^ These findings suggest that BDNF–TrkB signaling has a role in the behavioral abnormalities observed after METH administration.

Withdrawal from repeated use of stimulants, including METH and amphetamine, causes neurochemical alterations in the NAc, resulting in a depression-like behavior in rodents. Repeated treatment with amphetamine (or METH) increases the number of dendritic branches and the density of dendritic spines in the NAc. Furthermore, exposure to amphetamine produces a long-lasting increase in the length of dendrites, density of dendritic spines and number of branched spines in the NAc.^26, 27^ Thus, the ability of stimulants to alter the patterns of synaptic connectivity in the NAc may contribute to some of the long-term behavioral consequences after withdrawal from repeated amphetamine (or METH) use.^21, 28^ Accumulating evidence suggests a key role of BDNF–TrkB signaling in the structural plasticity of the ventral tegmental area (VTA)–NAc circuit in an addiction model.^21^ However, the exact molecular mechanisms by which BDNF–TrkB signaling mediates structural plasticity of the VTA–NAc circuit remain unknown.

The present study was performed to determine whether BDNF–TrkB signaling in the VTA–NAc circuit has a role in the pathophysiology of METH withdrawal symptoms. First, we examined whether withdrawal from repeated METH administration caused a depression-like behavior in mice. Second, we examined whether BDNF levels were altered in the brain after withdrawal from repeated METH administration. Third, we examined the effects of the TrkB antagonist ANA-12^23, 24, 29, 30^ and the TrkB agonist 7,8-DHF^23, 24, 30, 31^ on behavioral abnormalities (for example, depression and behavioral sensitization) and dendritic changes in the VTA–NAc circuit after METH withdrawal.

## Materials and methods

### Animals

Male adult C57BL/6 mice (8 weeks old, body weight 20–25 g at the beginning of experiments; Japan SLC, Hamamatsu, Japan) were housed under controlled temperature and 12 h light/dark cycles (lights on between 0700–1900 hours), with *ad libitum* food and water. All experiments were carried out in accordance with the Guideline for Animal Experimentation of Chiba University. This study was approved by the Chiba University Institutional Animal Care and Use Committee.

### Drugs

METH hydrochloride (Dainippon Pharmaceutical, Osaka, Japan) was dissolved in physiological saline, and METH (1.0 or 3.0 mg kg^−1^ expressed as a hydrochloride salt) was injected subcutaneously (s.c). at a volume of 10 ml kg^−1^. 7,8-DHF (Tokyo Chemical Industry, Tokyo, Japan) and ANA-12 (*N*-[2-[[(Hexahydro-2-oxo-1H-azepin-3-yl)amino]carbonyl]phenyl]-benzo[b]thiophene-2-carboxamide) (Maybridge, Loughborough, Leicestershire, UK) were dissolved in phosphate-buffered saline containing 17% dimethylsulfoxide. 7,8-DHF or ANA-12 was injected i.p. at a volume of 10 ml kg^−1^ body weight. Ketamine hydrochloride (10 mg kg^−1^; Daiichi-Sankyo Pharmaceutical, Tokyo, Japan) and paroxetine (Sigma-Aldrich, St Louis, MO, USA) were dissolved in the physiological saline. The experimental groups were not randomized in this study. Other chemicals were purchased from commercial sources.

### Behavioral tests

**Figure 1:**
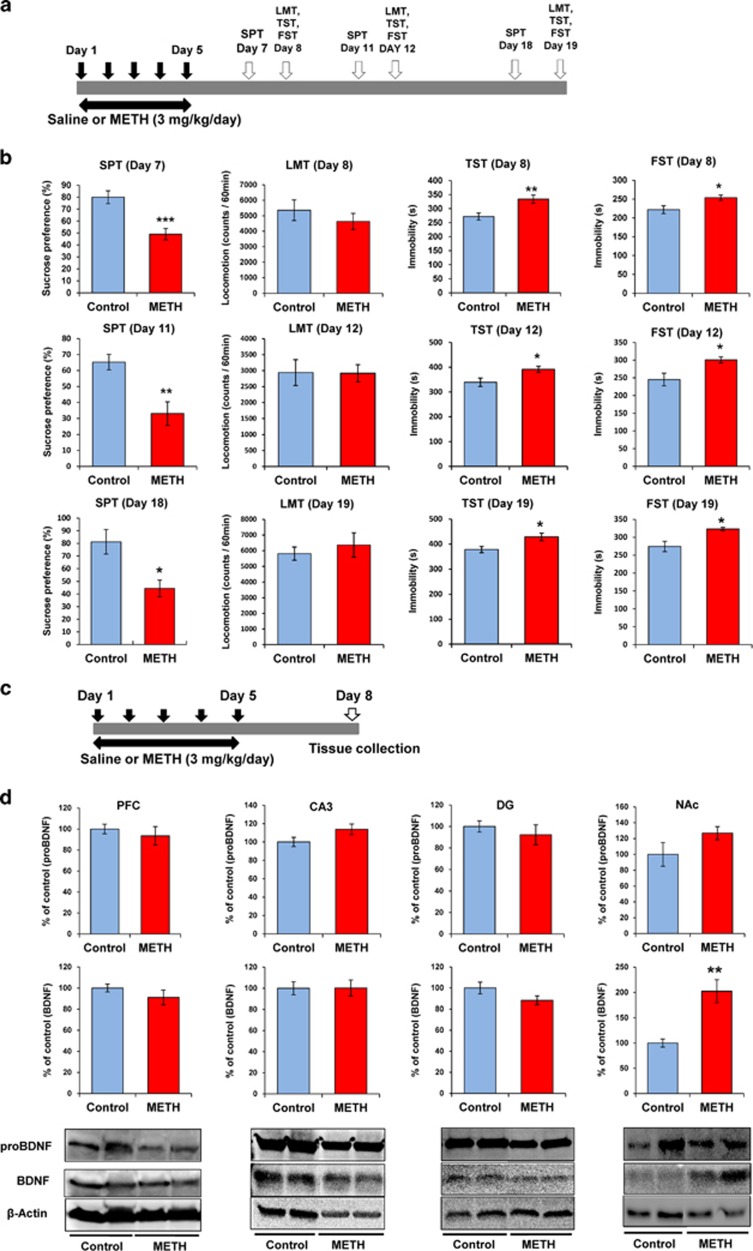
Depression-like behavior and levels of proBDNF and BDNF in the brain regions after withdrawal from repeated METH exposure. (**a**) Schedule of treatment and behavioral tests. Saline (10 ml kg^−1^ per day for 5 days) or METH (3 mg kg^−1^ per day for 5 days) was injected into mice. Behavioral tests were performed at days 7, 11 and 18 (SPT), and days 8, 12 and 19 (LMT, TST, FST). (**b**) SPT: sucrose preference of METH-treated mice was significantly lower than that of control (saline-treated) mice. LMT: there were no differences between control and METH-treated mice. TST and FST: the immobility time of METH-treated mice was significantly higher than that of control mice. *P<0.05, **P<0.01, ***P<0.001 as compared with control (saline-treated) group. Each value is the mean±s.e.m. (*n*=7–10 per group). (**c**) Schedule of treatment and western blot analysis. (**d**) Levels of proBDNF and BDNF (mature form) in the PFC, DG and CA3 of hippocampus had no difference between the two groups. Levels of BDNF in the NAc of METH-treated mice were significantly higher than those of control mice. Each value is the mean±s.e.m. (*n*=5–8 per group). **P<0.01 as compared with control (saline-treated) group. BDNF, brain-derived neurotrophic factor; DG, dentate gyrus; FST, forced swimming test; LMT, locomotion test; METH, methamphetamine; NAc, nucleus accumbens; PFC, prefrontal cortex; SPT, sucrose preference test; TST, tail suspension test.

**Figure 2:**
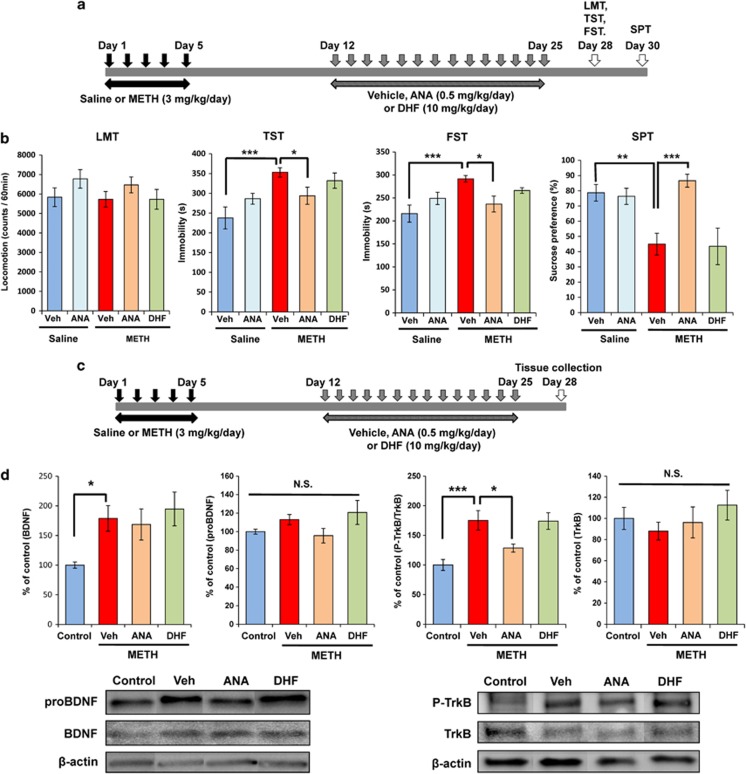
Effects of ANA-12 and 7,8-DHF on depression-like behavior in mice after withdrawal from repeated METH exposure. (**a**) Schedule of treatment and behavioral tests. Saline (10 ml kg^−1^ per day for 5 days) or METH (3 mg kg^−1^ per day for 5 days) was injected into mice from day 1 to day 5. After 1-week withdrawal, vehicle (10 ml kg^−1^ per day), ANA-12 (ANA: 0.5 mg kg^−1^ per day) or 7,8-DHF (DHF: 10 mg kg^−1^ per day) was administered for 14 days (days 12–25). Behavioral tests were performed at days 28 (LMT, TST, FST) and 30 (SPT). (**b**) LMT: there were no differences among the five groups. TST and FST: increased immobility time of METH-treated mice was significantly attenuated by subsequent repeated administration of ANA-12, but not 7,8-DHF. SPT: decreased sucrose preference of METH-treated mice was significantly attenuated by subsequent repeated administration of ANA-12, but not 7,8-DHF. Each value is the mean±s.e.m. (*n*=6–8 per group). *P<0.05, **P<0.01, ***P<0.001 as compared with METH+vehicle group. (**c**) Schedule of treatment and tissue collection. Saline (10 ml kg^−1^ per day for 5 days) or METH (3 mg kg^−1^ per day for 5 days) was injected into mice from day 1 to day 5. After 1-week withdrawal, vehicle (10 ml kg^−1^ per day), ANA-12 (ANA: 0.5 mg kg^−1^ per day) or 7,8-DHF (DHF: 10 mg kg^−1^ per day) was administered for 14 days (days 12–25). Brain samples were collected at day 28. (**d**) Increased levels of BDNF in the NAc were not altered by subsequent repeated administration of ANA-12 or 7,8-DHF. Increased levels of p-TrkB/TrkB ratio were significantly attenuated by subsequent repeated administration of ANA-12, but not 7,8-DHF. Each value is the mean±s.e.m. (*n*=5 or 6 per group). *P<0.05, ***P<0.001 as compared with METH+vehicle group. BDNF, brain-derived neurotrophic factor; FST, forced swimming test; LMT, locomotion test; METH, methamphetamine; NAc, nucleus accumbens; NS, not significant; SPT, sucrose preference test; TrkB, tropomyosin-related kinase; TST, tail suspension test.

Saline (10 ml kg^−1^ per day) or METH (3.0 mg kg^−1^ per day for 5 days) was administered into mice (day 1–day 5). Subsequently, behavioral tests were performed (Figures 1a, 2a and 5a).

#### Locomotion

Locomotor activity of mice was counted by the SCANET MV-40 (MELQUEST, Toyama, Japan), and cumulative exercise was recorded for 60 min. Cages were cleaned between testing session.

#### Tail suspension test

The mice were taken from their home cage and a small piece of adhesive tape was placed ~2 cm from the tip of their tail. A single hole was punched in the tape and mice were hung individually, on a hook. The immobility time of each mouse was recorded for 10 min. Mice were considered immobile only when they hung passively and completely motionless.

#### Forced swimming test

The mice were placed individually in a cylinder (diameter: 23 cm; height: 31 cm) containing 15 cm of water, maintained at 23±1 °C. Animals were tested in an automated forced-swim apparatus using SCANET MV-40. Immobility time was calculated from activity time as (total)−(active) time, using the apparatus analysis software. Cumulative immobility time was scored for 6 min during the test.

#### Sucrose preference test

Mice were habituated to a 1% sucrose solution for 24 h before the test day. Mice were deprived of water and food for 4 h from 1300 to 1700 hours, followed by a preference test spanning 1 h with water and 1% sucrose, delivered from identical bottles. The bottles containing water and sucrose were weighed at the before and end of this period and the sucrose preference (%) was determined.

#### Behavioral sensitization

**Figure 3:**
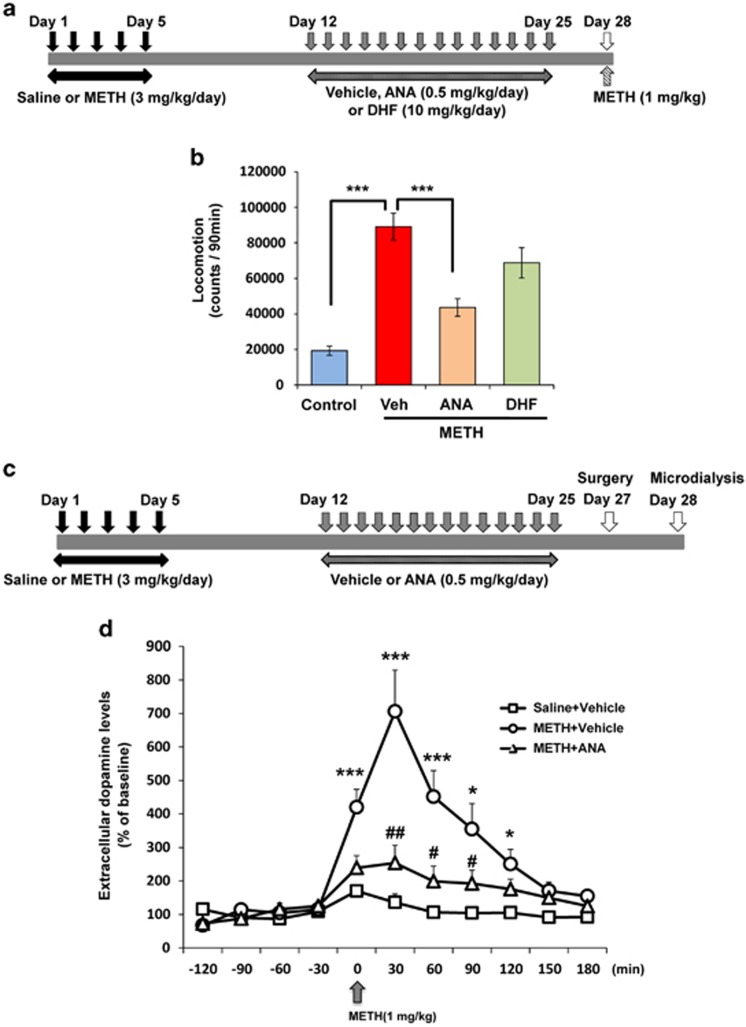
Effects of ANA-12 and 7,8-DHF on behavioral sensitization in mice after withdrawal from repeated METH exposure. (**a**) Schedule of treatment and behavioral test. Saline (10 ml kg^−1^ per day for 5 days) or METH (3 mg kg^−1^ per day for 5 days) was injected into mice from day 1 to day 5. After 1-week withdrawal, vehicle (10 ml kg^−1^ per day), ANA-12 (ANA: 0.5 mg kg^−1^ per day) or 7,8-DHF (DHF: 10 mg kg^−1^ per day) was administered for 14 days (days 12–25). Behavioral tests (METH-induced hyperlocomotion) were performed at day 28. (**b**) The established behavioral sensitization after the repeated METH (3 mg kg^−1^ per day for 5 days) exposure was significantly attenuated by subsequent repeated administration of ANA-12, but not 7,8-DHF. Each value is the mean±s.e.m. (*n*=7–9 per group). ***P<0.001 as compared with METH+vehicle group. (**c**) Schedule of treatment and *in vivo* microdialysis. Saline (10 ml kg^−1^ per day for 5 days) or METH (3 mg kg^−1^ per day for 5 days) was injected into mice from day 1 to day 5. After 1-week withdrawal, vehicle (10 ml kg^−1^ per day) or ANA-12 (ANA: 0.5 mg kg^−1^ per day) was administered for 14 days (days 12–25). Surgery was performed at day 27, and *in vivo* microdialysis was performed at day 28. (**d**) A marked increase of dopamine in the NAc shell after a METH (1 mg kg^−1^) injection of METH (3 mg kg^−1^ per day for 5 days)-treated mice was significantly attenuated by subsequent repeated administration of ANA-12. Each value is the mean±s.e.m. (*n*=5 or 6 per group). *P<0.05, ***P<0.001 as compared with control (saline+vehicle treated) group. ^#^*P*<0.05, ^##^*P*<0.01 as compared with METH+vehicle group. DHF, dihydroxyflavone; METH, methamphetamine.

Saline (10 ml kg^−1^ per day) or METH (3.0 mg kg^−1^ per day for 5 days) was administered into mice (day 1–day 5). Subsequently, vehicle (10 ml kg^−1^ per day), ANA-12 (0.5 mg kg^−1^ per day) or 7,8-DHF (10 mg kg^−1^ per day) was administered into mice from day 12 to 25 (Figure 3a). On day 28, all mice were given a low dose of METH (1.0 mg kg^−1^, s.c.), and locomotion was measured for 90 min, as reported previously.^32^

### Western blot analysis

Western blot analysis was performed as previously reported.^30, 33^ Saline (10 ml kg^−1^ per day) or METH (3.0 mg kg^−1^ per day for 5 days) was administered into mice (day 1–day 5). On day 8 (Figure 1c) or day 28 (Figure 2c), mice were killed, and prefrontal cortex (PFC), CA3 and dentate gyrus (DG) of hippocampus, and NAc were microdissected on ice, and stored at −80 °C. Tissue samples were homogenized in Laemmli lysis buffer. Equal amount of proteins (10–20 μg) for each sample were measured by DC protein assay kit (Bio-Rad, Hercules, CA, USA), and incubated for 5 min at 95 °C with protein buffer (125 mM Tris/HCl (pH 6.8), 20% glycerol, 0.1% bromophenol blue, 10% β-mercaptoethanol, 4% sodium dodecyl sulfate). The protein samples were loaded into AnykD mini-gels (Mini-PROTEAN TGX Precast Gel; Bio-Rad) for electrophoresis. Polyvinylidene difluoride membranes with transferred proteins were blocked with 2% BSA plus 5% nonfat dry milk in TBST (TBS+0.1% Tween-20; for BDNF and proBDNF) or 2% BSA in PBST (PBS+0.1% Tween-20; for GluA1) for 1 h and kept with primary antibodies overnight at 4 °C. The following primary antibody was used: proBDNF (1:400, Alomone Labs, Jerusalem, Israel), BDNF (1:200, H-117, Santa Cruz Biotechnology, Danvers, CA, USA), phosphorylated-TrkB (Tyr 706) (1:200, Santa Cruz Biotechnology), TrkB (80E3) (1:1000, Cell Signaling Technology, Danvers, MA, USA), GluA1 (1 μg ml^−1^, Abcam, Cambridge, UK) and β-actin (1:10 000, Sigma-Aldrich). The next day, blots were washed three times in TBST (or PBST) and incubated with horseradish peroxidase-conjugated anti-rabbit antibody or anti-mouse antibody for 1 h. After final three washes with TBST (or PBST), bands were detected using enhanced chemiluminescence plus the Western Blotting Detection system (GE Healthcare Bioscience, Tokyo, Japan) and the images were captured with Fuji LAS3000-mini imaging system (Fujifilm, Tokyo, Japan), and immunoreactive bands were quantified. The bands for proBDNF and BDNF (mature form) were confirmed using the brain samples from BDNF KO rat (SAGE Labs, Boyertown, PA, USA) (data not shown).

### Golgi staining

**Figure 4:**
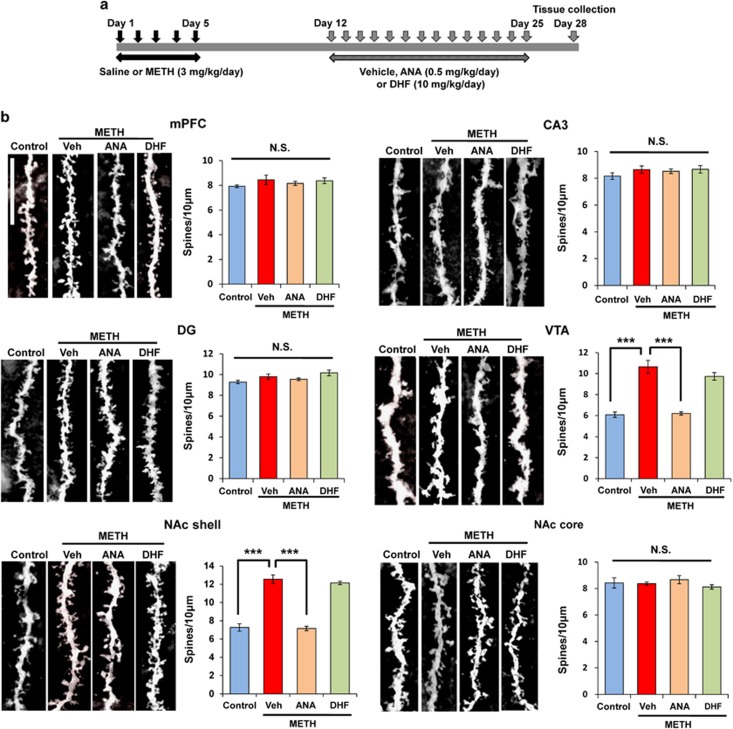
Effects of ANA-12 on dendritic changes in the brain regions after withdrawal from repeated METH exposure. (**a**) Schedule of treatment and sample collection. Saline (10 ml kg^−1^ per day for 5 days) or METH (3 mg kg^−1^ per day for 5 days) was injected into mice from day 1 to day 5. After 1-week withdrawal, vehicle (Veh: 10 ml kg^−1^ per day, i.p.), ANA-12 (ANA: 0.5 mg kg^−1^ per day, i.p.) or 7,8-DHF (DHF: 10 mg kg^−1^ per day, i.p.) were administered for 14 days (days 12–25). Sample collection for Golgi staining was performed at days 28. (**b**) PFC, DG, CA3, NAc core: there were no differences among the five groups. NAc shell and VTA: increased spine density in the NAc shell and VTA of METH-treated mice were significantly attenuated by subsequently repeated administration of ANA-12, but not 7,8-DHF. Each value is the mean±s.e.m. (*n*=5 or 6 per group). ***P<0.001 as compared with METH+vehicle group. DG, dentate gyrus; DHF, dihydroxyflavone; METH, methamphetamine; NAc, nucleus accumbens; NS, not significant; PFC, prefrontal cortex; VTA, ventral tegmental area.

Golgi staining was performed using the FD Rapid GolgiStain Kit (FD Neuro Technologies, Columbia, MD, USA), following the manufacturer's instructions as previously reported.^30, 33^ Saline (10 ml kg^−1^ per day) or METH (3.0 mg kg^−1^ per day for 5 days) was administered into mice (day 1–day 5). Subsequently, vehicle (10 ml kg^−1^ per day), ANA-12 (0.5 mg kg^−1^ per day) or 7,8-DHF (10 mg kg^−1^ per day) was administered into mice from day 12 to day 25 (Figure 4a). On day 28, all mice were deeply anesthetized using CO_2_ vapor from dry ice, and brains were removed from the skull and rinsed in double distilled water. Brains were immersed in the impregnation solution, made by mixing equal volumes of Solution A and B, overnight and then stored in fresh solution for 2 weeks in the dark. Brains were transferred into Solution C overnight and then stored in fresh solution at 4 °C for 1 week in the dark. Coronal brain sections (100-μm thickness) were cut on a cryostat (3050S, Leica Microsystems, Wetzlar, Germany), with the chamber temperature set at −20 °C. Each section was mounted in Solution C, on saline-coated microscope slides. After absorption of excess solution, sections were dried naturally, at room temperature. Dried sections were processed following the manufacturer's instructions. Briefly, images of dendrites within PFC, CA3 and DG of the hippocampus, NAc and VTA were captured using a × 100 objective with a Keyence BZ-9000 Generation microscope (Keyence, Osaka, Japan). Spines were counted along PFC, CA3, DG, NAc and VTA dendrites starting from their point of origin from the primary dendrite. For spine density measurements, all clearly evaluable areas containing 50–100 μm of secondary dendrites from each imaged neuron were used. To determine relative spine density, spines on multiple dendritic branches from a single neuron were counted to obtain an average spine number per 10 μm, in a blind manner. For spine number measurements, only spines that emerged perpendicular to the dendritic shaft were counted. Three neurons per section, six sections per animal (*n*=5 or 6) were analyzed. The average value for each region, in each individual was obtained. These individual averages were then combined to yield a grand average for each region.

### Bilateral infusion of ANA-12 into NAc shell and NAc core

**Figure 5:**
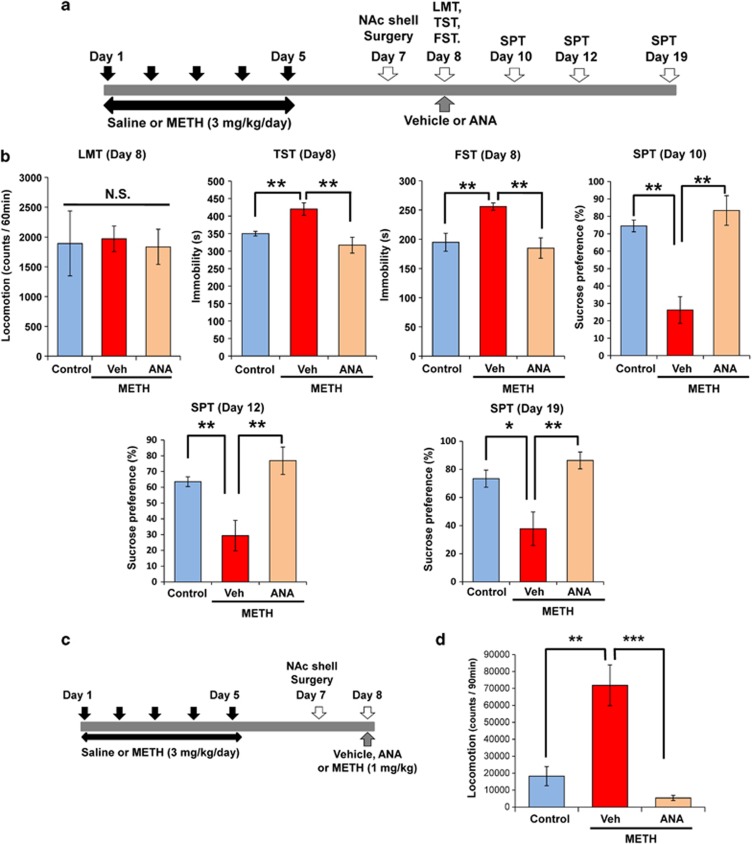
Effects of bilateral injection of ANA-12 into NAc shell on depression-like behavior and behavioral sensitization after withdrawal from repeated METH exposure. (**a**) Schedule of treatment and behavioral tests. Saline (10 ml kg^−1^ per day for 5 days) or METH (3 mg kg^−1^ per day for 5 days) was injected into mice from day 1 to day 5. Two days after the final injection, surgery was performed as described in the Materials and Methods section. Vehicle or ANA-12 was injected bilaterally into the NAc shell. Behavioral tests were performed 1 h (LMT), 3 h (TST), 5 h (FST) after injection of ANA-12. Then behavioral tests were performed at days 10, 12 and 19 (SPT). (**b**) LMT: there were no differences among the three groups. TST and FST: increased immobility time of METH-treated mice was significantly attenuated by a single bilateral injection of ANA-12 into the NAc shell. SPT: decreased sucrose preference of METH-treated mice was significantly improved after a single bilateral injection of ANA-12 into the NAc shell. Antidepressant effect of ANA-12 showed a long-lasting (11 days after ANA-12 injection). Each value is the mean±s.e.m. (*n*=5–7 per group). *P<0.05, **P<0.01 as compared with METH+vehicle group. (**c**) Schedule of treatment and behavioral test. Saline (10 ml kg^−1^ per day for 5 days) or METH (3 mg kg^−1^ per day for 5 days) was injected into mice from day 1 to 5. Two days after the final injection, surgery was performed as described in the Materials and Methods section. Vehicle or ANA-12 was injected bilaterally into the NAc shell. METH (1 mg kg^−1^, s.c.) was administered into all mice 60 min after injection of ANA-12 into NAc. (**d**) Locomotion was counted for 90 min. Each value is the mean±s.e.m. (*n*=5–7 per group). **P<0.01, ***P<0.001 as compared with METH+vehicle group. BDNF, brain-derived neurotrophic factor; FST, forced swimming test; LMT, locomotion test; METH, methamphetamine; NAc, nucleus accumbens; NS, not significant; SPT, sucrose preference test; TST, tail suspension test.

Surgery and bilateral injection of ANA-12 into NAc shell and core was performed as reported previously.^30^ Saline (10 ml kg^−1^ per day) or METH (3.0 mg kg^−1^ per day for 5 days) was administered into mice (day 1–day 5). On day 7, all mice were anesthetized with pentobarbital (50 mg kg^−1^), and placed in a stereotaxic frame (Figures 5a and c). Microinjection probes were placed bilaterally into the NAc shell (+1.7 mm anterior/posterior (AP), ±0.75 mm medial/lateral (ML) from the bregma and −3.6 mm dorsal/ventral (DV) with respect to the dura), and NAc core (+1.54 mm AP, ±0.75 mm ML from the bregma and −3.0 mm DV with respect to the dura), according to mouse brain atlas.^34^ Probes were secured onto the skull using stainless-steel screws and dental acrylic. ANA-12 (0.1 nmol l^−1^, 0.1 μl min^−1^ for 5 min) or vehicle was injected bilaterally in 23 h after surgery (day 8). Behavioral evaluation was performed 1 h (locomotion), 3 h (tail suspension test; TST) and 5 h (forced swimming test; FST) after a single infusion of ANA-12.

### *In vivo* microdialysis study

Saline (10 ml kg^−1^ per day) or METH (3.0 mg kg^−1^ per day for 5 days) was administered into mice (day 1–day 5). Subsequently, vehicle (10 ml kg^−1^ per day), or ANA-12 (0.5 mg kg^−1^ per day) was administered into mice from day 12 to day 25 (Figure 3c). On day 27, mice were anesthetized with sodium pentobarbital (50 mg kg^−1^) prior to the stereotaxic implantation of a probe into the NAc shell (+1.7 mm AP, ±0.75 mm ML from the bregma and −3.6 mm DV with respect to the dura), according to mouse brain atlas.^34^ Probes were secured onto the skull using stainless-steel screws and dental acrylic. Twenty-four hours (day 28) after surgery, *in vivo* microdialysis was performed at conscious and free-moving mice. Probes were perfused continuously with artificial cerebrospinal fluid (147 mM NaCl, 4 mM KCl, 2.3 mM CaCl_2_) at a rate of 2 μl min^−1^. The dialysate was collected in 30-min fractions, and METH (1 mg kg^−1^, s.c.) was administered into all mice. Dopamine levels in each fraction were measured by high performance liquid chromatography with electrochemical detection, using a reversed phase column (EICOMPAK PP-ODS II, 4.6 × 30 mm, Eicom, Kyoto, Japan) and a mobile phase 2% MeOH/100 mM phosphate buffer (pH 5.4) containing 50 mg l^−1^ disodium EDTA and 500 mg l^−1^ sodium decane-1-sulfonate.^23, 32^ Four samples were analyzed to establish baseline levels of extracellular dopamine, before the administration of METH.

### Statistical analysis

Data were presented as the mean±s.e.m. The data were analyzed by Student's *t*-test or one-way analysis of variance (ANOVA) or two-way ANOVA, followed *post hoc* the Fisher least significant difference test. The data of *in vivo* microdialysis were analyzed by repeated ANOVA, followed *post hoc* the Fisher least significant difference test. The *P*-values<0.05 were considered statistically significant.

## Results

### Depression-like behaviors induced by METH withdrawal

First, we examined whether the repeated administration of METH (3 mg kg^−1^ per day for 5 days) caused a depression-like behavior in mice. In the TST and FST, the immobility time of the METH-treated mice was significantly higher than that of control mice at days 8, 12 and 19 (Figures 1a and b). In the sucrose preference test (SPT), sucrose preference (%) in the METH-treated group was significantly lower than that of the control group at days 7, 11 and 18 (Figures 1a and b). In contrast, spontaneous locomotion was similar in both groups (Figures 1a and b). This data showed that repeated administration of METH caused a long-lasting (>14 days after the last METH dosing) depression-like behavior in mice.

### Levels of BDNF and its precursor proBDNF in the brain regions after repeated METH administration

Because BDNF has a key role in the depression-like behavior, we measured the levels of BDNF (mature form) and its precursor proBDNF protein in the brains of mice treated with METH (3 mg kg^−1^ per day for 5 days). The levels of proBDNF in the METH-treated mice were not different from those of the control mice (Figures 1c and d). In contrast, in the METH-treated group, the levels of BDNF in the NAc were significantly (*t*=3.925, *P*<0.01) higher than those of the control group (Figure 1d). The levels of BDNF were similar in both groups in other brain regions, including the DG and the CA3 field of the hippocampus, and the PFC (Figures 1c and d).

### Effects of ANA-12 or 7,8-DHF on depressive-like behavior and TrkB phosphorylation after METH withdrawal

First, we examined whether a single administration of the TrkB antagonist ANA-12 and the TrkB agonist 7,8-DHF could affect behavioral abnormalities (depression and behavioral sensitization) in mice after repeated METH administration. A single administration of ANA-12 (0.5 mg kg^−1^) or 7,8-DHF (10 mg kg^−1^) did not alter the depression-like behavior (Supplementary Figures S1A and S1B) or the behavioral sensitization (Supplementary Figures S1C and S1D) in mice after repeated administration of METH (3 mg kg^−1^ per day for 5 days).

Next, we examined whether the subchronic administration of ANA-12 or 7,8-DHF in mice could correct depression induced by repeated METH administration. After repeated METH (3 mg kg^−1^ per day for 5 days) administration followed by 1-week withdrawal, the mice were administered vehicle (10 ml kg^−1^ per day for 14 days), ANA-12 (0.5 mg kg^−1^ per day for 14 days) or 7,8-DHF (10 mg kg^−1^ per day for 14 days). Behavioral tests for depression-like behavior were performed 3 and 5 days after the end of the treatment (Figure 2a). One-way ANOVA revealed statistical differences among the five groups (locomotion: F_(4,41)_=1.072, *P*=0.383; TST: F_(4,38)_=5.823, *P*=0.001; FST: F_(4,31)_=3.796, *P*=0.013; SPT: F_(4,37)_=7.581, *P*<0.001). Subchronic administration of ANA-12, but not 7,8-DHF, significantly improved the increased immobility time of TST and FST, and the decreased sucrose preference of SPT in mice after repeated METH administration (Figure 2b).

The same experiment was performed a second time, brain samples were obtained at day 28 (Figure 2c). Subsequently, we performed western blot analysis of BDNF, proBDNF, TrkB and p-TrkB in NAc. Treatment with ANA-12 or 7,8-DHF did not alter increased levels of BDNF in the NAc of METH-treated mice (Figure 2d). In contrast, treatment with ANA-12, but not 7,8-DHF, significantly attenuated an increase of p-TrkB/TrkB ratio in the NAc of METH-treated mice (Figure 2d). One-way ANOVA revealed statistical differences among the four groups (p-TrkB/TrkB ratio: F_(3,18)_=9.315, *P*<0.001). There were no differences for proBDNF and TrkB among the four groups.

### Effects of ANA-12 or 7,8-DHF on behavioral sensitization after METH withdrawal

Next, we examined whether the subchronic administration of ANA-12 or 7,8-DHF in mice could correct the behavioral sensitization induced by repeated METH administration. After repeated METH (3 mg kg^−1^ per day for 5 days) administration followed by 1-week withdrawal, the mice were administered vehicle (10 ml kg^−1^ per day for 14 days), ANA-12 (0.5 mg kg^−1^ per day for 14 days) or 7,8-DHF (10 mg kg^−1^ per day for 14 days). Behavioral test was performed 3 days after the end of the treatment. The mice were injected METH (1 mg kg^−1^, s.c.) at day 28 (Figure 3a). One-way ANOVA revealed a significant difference among the four groups (F_(3,27)_=19.49, *P*<0.001). Behavioral sensitization induced by the repeated administration of METH was significantly (*P*<0.001) improved by subsequent subchronic administration of ANA-12, but not 7,8-DHF (Figure 3b).

The same experiment was performed a second time. After the repeated METH (3 mg kg^−1^ per day for 5 days) administration and 1-week withdrawal, mice were administered vehicle (10 ml kg^−1^ per day for 14 days) or ANA-12 (0.5 mg kg^−1^ per day for 14 days). Surgery was performed 2 days (day 27) after the final administration, followed by *in vivo* microdialysis at free-moving mice 1 day after the surgery. Then METH (1 mg kg^−1^, s.c.) was administered to all mice (day 28) (Figure 3c). Repeated ANOVA analysis revealed a significant difference among the three groups (F_(2,13)_=13.76, *P*<0.001). An increase of extracellular dopamine in the NAc shell of METH-treated mice after a single administration of METH (1 mg kg^−1^) was significantly attenuated by subsequent repeated administration of ANA-12 (Figure 3d).

These findings suggest that behavioral abnormalities (depression and behavioral sensitization) in mice after METH withdrawal were improved by subsequent repeated administration of the TrkB antagonist ANA-12, but not TrkB agonist 7,8-DHF.

### Lack of effects of ketamine and paroxetine on depressive-like behavior after METH withdrawal

The *N*-methyl-D-aspartate receptor antagonist ketamine is the most attractive drug for treating treatment-resistant depression.^35, 36, 37, 38^ A single administration of ketamine (10 mg kg^−1^, i.p.) confers a rapid and long-lasting antidepressant effect in chronic mild stress models of depression.^39, 40^ Three days after the repeated administration of METH (3 mg kg^−1^ per day for 5 days), saline or ketamine (10 mg kg^−1^, i.p.) was administered (Supplementary Figure S2A). One-way ANOVA revealed statistical differences among the three groups (locomotion: F_(2,21)_=3.041, *P*=0.069; TST: F_(2,19)_=5.801, *P*=0.011; FST: F_(2,18)_=5.09, *P*=0.018; SPT: F_(2,16)_=6.727, *P*=0.008)). However, *post hoc* analysis showed that a single administration of ketamine (10 mg kg^−1^) had no antidepressant effects in depressed mice after METH withdrawal (Supplementary Figure S2B).

Three days after the repeated administration of METH (3 mg kg^−1^ per day for 5 days), vehicle or the antidepressant paroxetine (10 mg kg^−1^ per day for 14 days) was administered (Supplementary Figure S2C). Two-way ANOVA revealed statistical differences among the four groups (locomotion: METH, F_(1,28)_=1.003, *P*=0.325; paroxetine, F_(1,28)_=1.102, *P*=0.303; interaction (METH × paroxetine) F_(1,28)_=0.295, *P*=0.591; TST: METH, F_(1,23)_=19.93, *P*<0.001; paroxetine, F_(1,23)_=7.075, *P*=0.014; interaction (METH × paroxetine), F_(1,23)_=0.074, *P*=0.789; FST: METH, F_(1,24)_=16.16, *P*=0.001; paroxetine, F_(1,24)_=0.272, *P*=0.607; interaction (METH × paroxetine), F_(1,24)_=4.188, *P*=0.520; SPT: METH, F_(1,27)_=16.49, *P*<0.001; paroxetine, F_(1,27)_=0.190, *P*=0.666; interaction (METH × paroxetine), F_(1,27)_=0.006, *P*=0.946). However, the *post hoc* analysis showed that subchronic administration of paroxetine (10 mg kg^−1^ per day for 14 days) had no antidepressant effects in depressed mice after METH withdrawal (Supplementary Figure S2D).

### Effects of repeated administration with ANA-12 and 7,8-DHF on dendritic spine alterations and synaptic protein levels in PFC, NAc, hippocampus and VTA

Repeated treatment with stimulants (for example, amphetamine, METH) is known to increase the number of dendritic branches and the density of dendritic spines in the NAc.^26, 27, 41^ In this study, we examined whether the subchronic administration of ANA-12 (0.5 mg kg^−1^ per day for 14 days) or 7,8-DHF (10 mg kg^−1^ per day for 14 days) had an effect on spine density and synaptic protein GluA1, a subtype of AMPA receptor, in different brain regions (Figure 4a and Supplementary Figure S3A). One-way ANOVA revealed significant differences among the four groups (mPFC: F_(3,16)_=0.956, *P*=0.438; CA3: F_(3,17)_=0.968, *P*=0.430; DG: F_(3,16)_=3.06, *P*=0.580; NAc shell: F_(3,18)_=74.76, *P*<0.001; NAc core: F_(3,16)_=0.689, *P*=0.572; VTA: F_(3,18)_=48.25, *P*<0.001). The *post hoc* analysis showed that the increase of spine density in the NAc shell and VTA of METH-treated mice was significantly attenuated by subsequent repeated administration of ANA-12, but not 7,8-DHF (Figure 4b).

One-way ANOVA of GluA1 data revealed statistical differences among the four groups (PFC: F_(3,21)_=0.578, *P*=0.636; CA3: F_(3,19)_=0.616, *P*=0.613; DG: F_(3,21)_=0.774, *P*=0.552; NAc: F_(3,23)_=6.012, *P*=0.004). The *post hoc* analysis showed that the increase of GluA1 protein in the NAc of METH-treated mice was significantly attenuated by subsequent repeated administration of ANA-12, but not 7,8-DHF (Supplementary Figures S3A and S3B).

### A single bilateral injection of ANA-12 into the NAc shell, but not NAc core, improves depressive-like behaviors and behavioral sensitization after METH withdrawal

The above data suggest that increased BDNF levels and spine density in the NAc have a role in the depression-like behavior in mice treated with METH (3 mg kg^−1^ per day for 5 days). We examined whether ANA-12 could attenuate the depressive-like behavior and behavioral sensitization in mice after METH withdrawal. A single bilateral injection of ANA-12 (0.1 nmol l^−1^, 0.1 μl min^−1^ for 5 min) into the NAc shell significantly (*P*<0.01) attenuated the increased immobility time of TST and FST, and significantly (*P*<0.01) increased the reduction in SPT (Figures 5a and b). Interestingly, a single bilateral injection of ANA-12 into NAc shell showed a long-lasting (>11 days after injection of ANA-12 into NAc shell) antidepressant effect (Figure 5b). Next, we examined the effect of ANA-12 on the established behavioral sensitization after METH withdrawal. Ninety minutes after a single bilateral infusion of ANA-12 (0.1 nmol l^−1^, 0.1 μl min^−1^ for 5 min) into the NAc shell, METH (1 mg kg^−1^, s.c.) was injected to all mice. A single bilateral injection of ANA-12 into the NAc shell also significantly (*P*<0.001) attenuated the behavioral sensitization in mice after METH withdrawal (Figures 5c and d).

In contrast, a single bilateral injection of ANA-12 (0.1 nmol l^−1^, 0.1 μl min^−1^ for 5 min) into the NAc core did not attenuate the increased immobility time of TST and FST, and the reduction in SPT of METH-treated mice (Supplementary Figures S4A and S4B). These findings suggest that NAc shell, but not NAc core, has a key role in the METH withdrawal symptoms.

## Discussion

The major findings of the present study are that increased BDNF–TrkB signaling in the NAc shell has a key role in the behavioral abnormalities in mice after repeated METH administration and that the TrkB antagonist ANA-12 is a potential therapeutic drug for METH withdrawal symptoms. BDNF levels in the NAc of METH-treated mice were significantly higher than those of the control mice, suggesting that increased BDNF levels in the NAc are involved in the depression-like behavior after METH withdrawal. Furthermore, blocking TrkB with ANA-12 in the NAc shell showed therapeutic effects on depression and behavioral sensitization after repeated METH exposure, confirming the role of the activated BDNF–TrkB signaling pathway in the mechanism of behavioral abnormalities after METH withdrawal. To the best of our knowledge, this is the first report which showed that that blocking BDNF–TrkB signaling in the NAc shell represents a potential therapeutic approach for behavioral abnormalities after repeated METH exposure. Previously, several studies had suggested that BDNF–TrkB signaling has a causal role in the plasticity observed in other abused drugs, including cocaine and morphine.^42, 43^ Taken together, these findings identify TrkB antagonists such as ANA-12 as promising therapeutic drugs for multiple withdrawal symptoms associated with METH abuse in humans.

Repeated administration of METH resulted in a marked increase of BDNF protein (not proBDNF) and p-TrkB/TrkB ratio in the NAc, resulting in the long-lasting depression-like behavior in mice after METH withdrawal. Considering the role of BDNF–TrkB signaling in the NAc in depression,^16, 30, 33, 44, 45, 46^ our findings suggest that a marked increase of BDNF within the NAc by repeated METH exposure contributes to long-lasting behavioral abnormalities (depression and behavioral sensitization) and that TrkB antagonists confer beneficial effects against these behavioral abnormalities by blocking TrkB signaling in the NAc shell, but not NAc core. Interestingly, we found that the *N*-methyl-D-aspartate receptor antagonist ketamine was not an effective antidepressant to treat the depression-like behavior after METH withdrawal. In chronic mild stress models of depression,^39, 40^ a single administration of ketamine confers a rapid and long-lasting antidepressant effect by increasing BDNF levels in the PFC and hippocampus. Because ketamine's antidepressant effect was not detected in the METH withdrawal model, it is unlikely that BDNF–TrkB signaling in the NAc may be involved in the antidepressant's mechanisms of ketamine.

Although some selective serotonin reuptake inhibitors (SSRIs), including paroxetine, have been studied as potential therapeutic drugs for METH dependence, SSRIs failed to distinguish themselves from placebo.^47, 48^ In this study, we observed that repeated treatment with paroxetine (10 mg kg^−1^ per day for 14 days) had no antidepressant effect on the depression-like behavior after METH withdrawal. Because an increase of BDNF in the hippocampus by chronic SSRI administration has been implicated in the mechanism of antidepressant action of SSRI,^11, 12, 15^ the lack of efficacy of paroxetine for METH-induced depression is of great interest. To our knowledge, there are no reports showing the beneficial effects of the current antidepressants in patients with METH withdrawal. Taken together, it is likely that TrkB antagonists are more suitable drugs than the current antidepressants for METH withdrawal symptoms.

Repeated exposure to METH results in a progressively enhanced and enduring behavioral response to the drug, a phenomenon known as behavioral sensitization. A number of behavioral, neurochemical, biochemical and molecular studies have shown that the initiation of this complex process involves the interaction of several neurotransmitters, neuropeptides, neurotrophic factors and their associated receptor signaling pathways.^49, 50^ Dopamine release and dopamine-induced behavioral abnormalities by METH have been reported to be significantly suppressed by pretreatment with intra-NAc injections of TrkB antibodies.^25^ In this study, we found that behavioral sensitization after repeated METH administration is improved after subsequent repeated administration of ANA-12 or a single bilateral infusion of ANA-12 into the NAc shell, but not NAc core. Furthermore, the *in vivo* microdialysis study showed that an increase of extracellular dopamine release in the NAc shell of METH-treated mice was significantly attenuated after subsequent repeated administration of ANA-12 or a single bilateral infusion of ANA-12 into the NAc shell. These findings add weight to the theory that BDNF–TrkB signaling in the NAc shell has a key role in METH-induced behavioral sensitization in rodents. Therefore, it is likely that blocking TrkB signaling in the NAc shell by a TrkB antagonist can attenuate the behavioral sensitization after repeated METH administration.

Deep brain stimulation is a neurosurgical intervention in which implanted electrodes deliver microelectrical pulses to target areas in the human brain. Deep brain stimulation has been used for treatment-refractory patients suffering from substance abuse and depression.^51, 52^ The NAc is known to mediate the rewarding and reinforcing properties of drugs of abuse and constitutes a key target in the treatment of depression, given that anhedonia is one of the key defining symptoms of depression.^53^ Interestingly, deep brain stimulation of the NAc shell, but not the NAc core, significantly attenuates cocaine priming-induced reinstatement of drug seeking in rodents.^54, 55^ In this study, we observed an increased spine density in the NAc shell, but not in the NAc core, of the METH-treated mice. Furthermore, an increase of the spine density in the NAc shell by repeated METH exposure could be attenuated by subsequent repeated administration of ANA-12. Moreover, a single bilateral infusion of ANA-12 into the NAc shell showed a long-lasting antidepressant effect for depression-like behaviors after METH withdrawal. Taken together, a bilateral infusion of TrkB antagonist into the NAc shell is a potential therapeutic approach for patients with severe substance abuse.

In conclusion, our study demonstrated that an increased BDNF–TrkB signaling in the NAc shell may be implicated in the long-lasting behavioral abnormalities (depression and behavioral sensitization) in mice after the repeated exposure to METH and that blockage of TrkB signaling by ANA-12 could attenuate these behavioral abnormalities after METH withdrawal. These findings make TrkB antagonists promising therapeutic agents for the treatment of multiple withdrawal symptoms associated with METH abuse.
