# Supplementary figures and images for: BDNF–TrkB signaling in the nucleus accumbens shell of mice has key role in methamphetamine withdrawal symptoms
Source: Transl Psychiatry. 2015 Oct 27;5(10):e666–. doi: 10.1038/tp.2015.157 (PMC4930133; doi:10.1038/tp.2015.157)

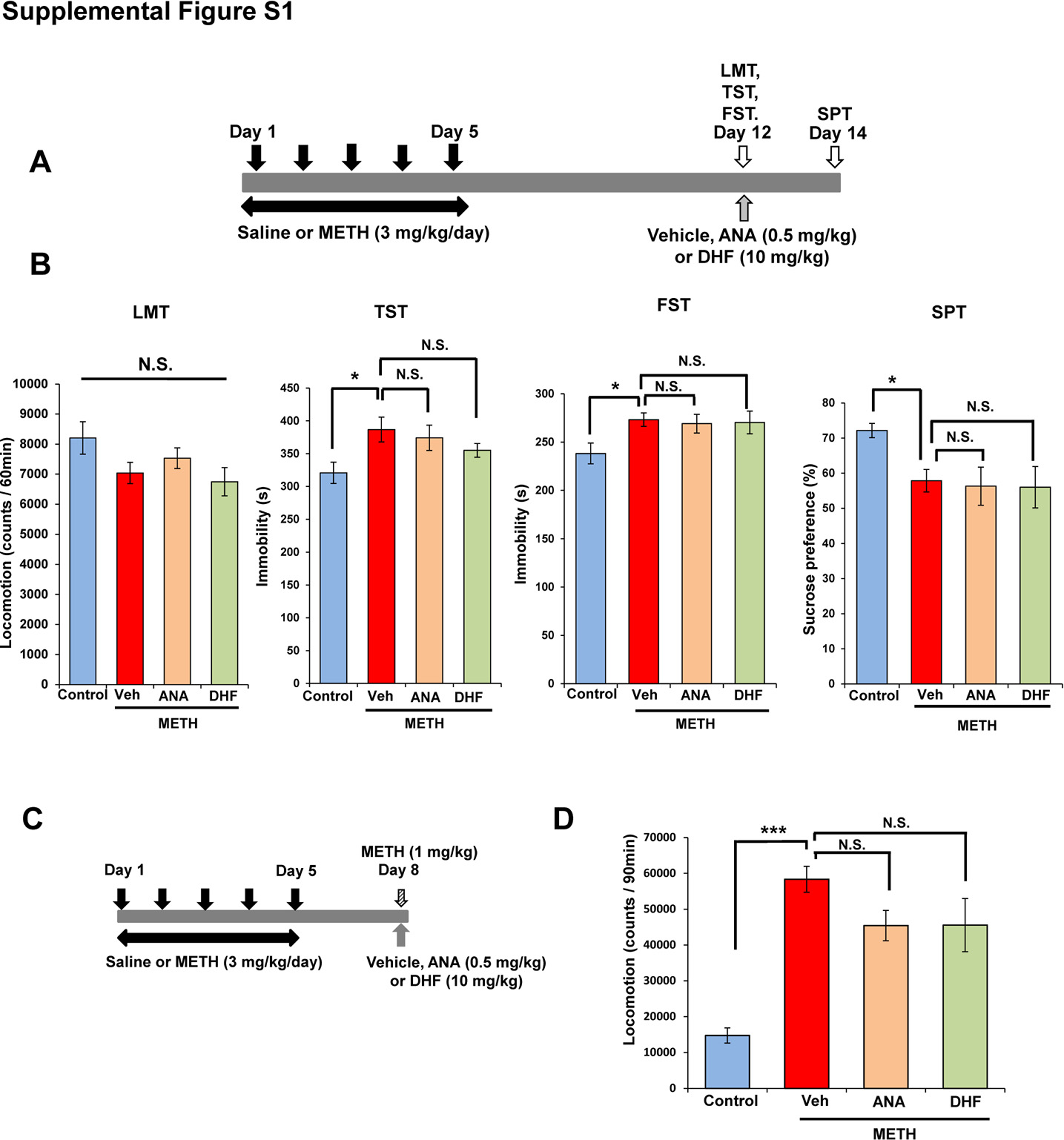

Supplement: Supplementary Figure S1 [file tp2015157x1.tif]

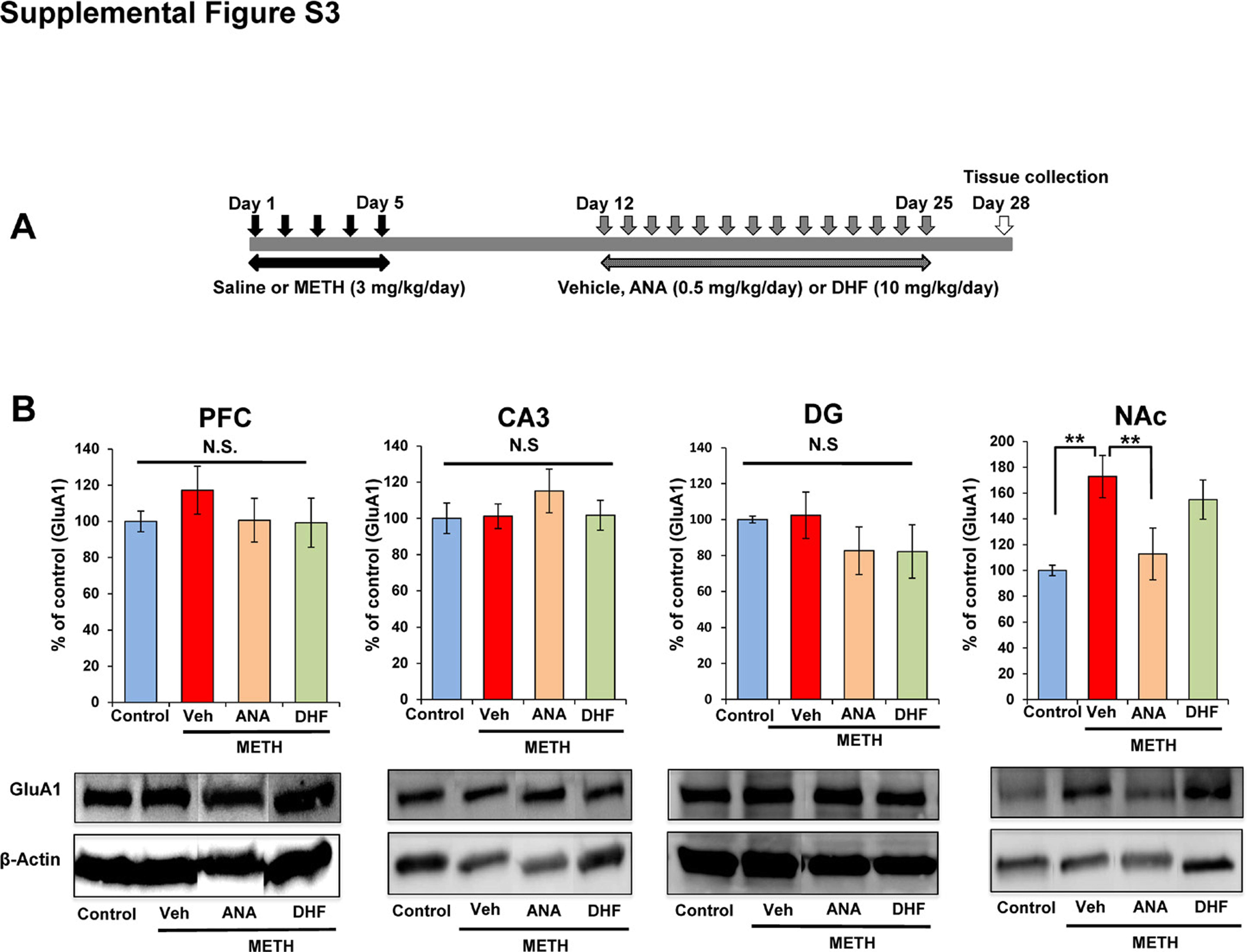

Supplement: Supplementary Figure S3 [file tp2015157x3.tif]

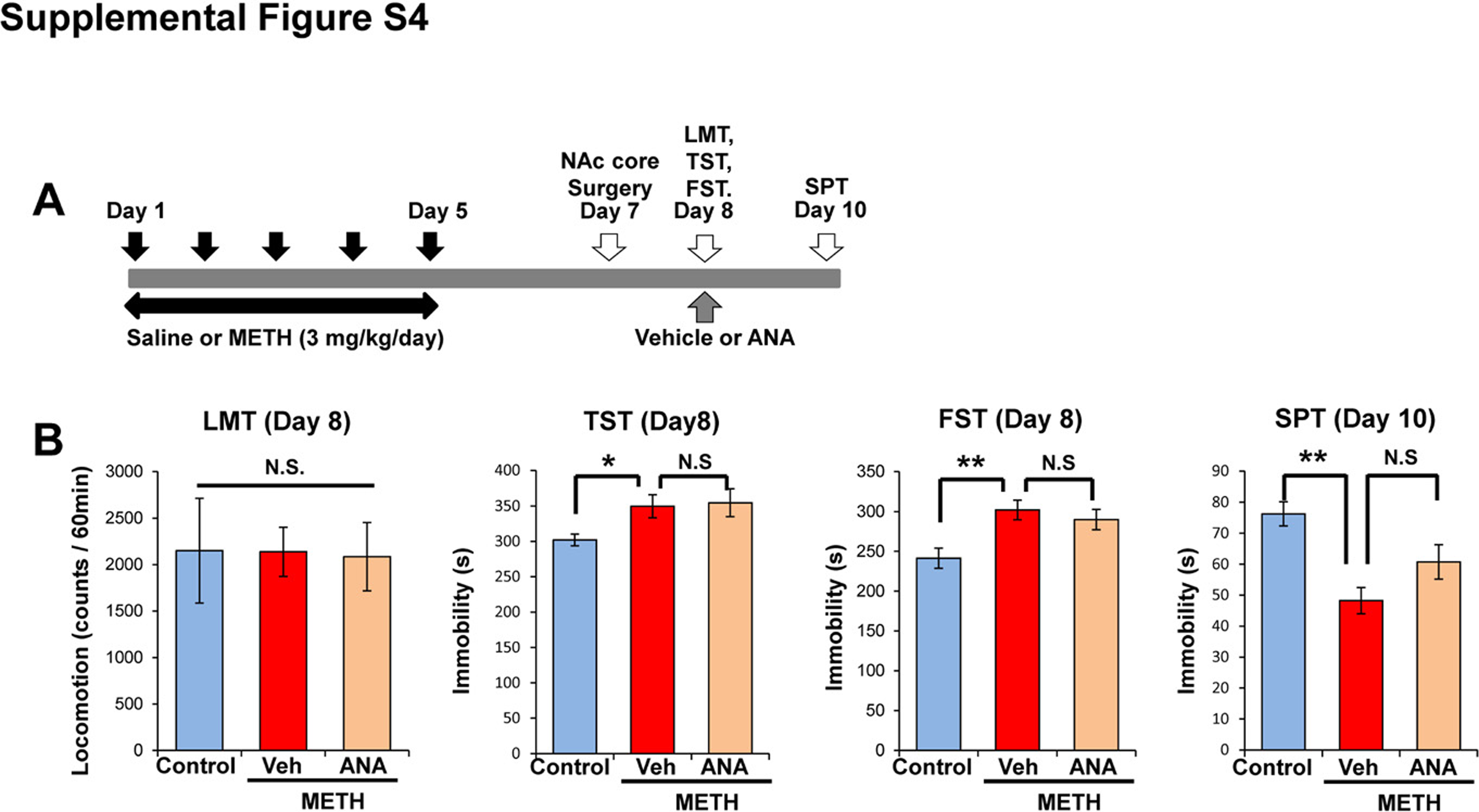

Supplement: Supplementary Figure S4 [file tp2015157x4.tif]
