# Supplementary material for: BDNF–TrkB signaling in the nucleus accumbens shell of mice has key role in methamphetamine withdrawal symptoms
Source: Transl Psychiatry. 2015 Oct 27;5(10):e666–. doi: 10.1038/tp.2015.157 (PMC4930133; doi:10.1038/tp.2015.157)
Supplement: Supplementary Figure Legends [file tp2015157x5.doc]

**Supplementary Information**

**Supplementary Figure S1. Lack of effects of ANA-12 and 7,8-DHF on depression-like behavior and behavioral sensitization in mice after withdrawal from repeated METH exposure**

A: Schedule of treatment and behavioral tests. Saline (10 ml/kg/day for 5 days) or METH (3 mg/kg/day for 5 days) were injected into mice from day 1 to day 5. After 1-week withdrawal, vehicle (10 ml/kg), ANA-12 (ANA: 0.5 mg/kg), or 7,8-DHF (DHF: 10 mg/kg) were administered i.p. (day 8). LMT, TST, FST were performed at 1, 3, and 5 hours after injection. SPT was performed at day 10. B: LMT: There were no differences among the four groups. TST and FST: Increased immobility time of METH-treated mice were not altered by subsequent single administration of ANA-12 or 7,8-DHF. SPT: Decreased sucrose preference of METH-treated mice was not altered by subsequent single administration of ANA-12 or 7,8-DHF. Each value is the mean ± S.E.M. (n = 6-8 per group). *P < 0.05, **P < 0.01, ***P < 0.001 as compared with METH + vehicle group. SPT: sucrose preference test, LMT: locomotion, TST: tail suspension test, FST: forced swimming test. N.S.: not significant. C: Schedule of treatment and behavioral test. Saline (10 ml/kg/day for 5 days) or METH (3 mg/kg/day for 5 days) were injected into mice from day 1 to day 5. Vehicle (10 ml/kg), ANA-12 (ANA: 0.5 mg/kg), or 7,8-DHF (DHF: 10 mg/kg) were administered i.p. (day 8). METH (1 mg/kg) was injected s.c. into all mice 0.5 hour after injection. D: The established behavioral sensitization after the repeated METH (3 mg/kg/day for 5 days) exposure was not altered by subsequent single administration of ANA-12 or 7,8-DHF. Each value is the mean ± S.E.M. (n = 7 or 8 per group). ***P < 0.001 as compared with METH + vehicle group. N.S.: not significant.

**Supplementary Figure S2. Lack of effects of ketamine and paroxetine on depression-like behavior in mice after withdrawal from repeated METH exposure**

A: Schedule of treatment and behavioral tests. Saline (10 ml/kg/day for 5 days) or METH (3 mg/kg/day for 5 days) were injected into mice from day 1 to day 5. Three days (day 8) after the final injection, vehicle (10 ml/kg, i.p.), or ketamine (10 mg/kg, i.p.) was administered. LMT, TST, and FST were performed at 1, 3, 5 hours after ketamine injection. SPT was performed at day 9. B: LMT: There were no differences among the three groups. TST and FST: Increased immobility time of METH-treated mice was not altered by a single administration of ketamine (10 mg/kg). SPT: Decreased sucrose preference of METH-treated mice was not altered by a single administration of ketamine. Each value is the mean ± S.E.M. (n = 6-9 per group). *P < 0.05, **P < 0.01, ***P < 0.001 as compared with control (saline-treated) group. N.S.: no significance. C: Schedule of treatment and behavioral test. Saline (10 ml/kg/day for 5 days) or METH (3 mg/kg/day for 5 days) were injected into mice from day 1 to day 5. Saline (Sal: 10 ml/kg/day, i.p.), or paroxetine (Par: 10 mg/kg/day, i.p.) were administered for 14 days (days 8-21). Behavioral tests were performed at days 28 (LMT, TST, FST) and 30 (SPT). D: LMT: There were no differences among the four groups. TST and FST: Increased immobility time of METH-treated mice was not altered by subsequent repeated administration of paroxetine (10 mg/kg/day for 14 days). SPT: Decreased sucrose preference of METH-treated mice was not altered by subsequent repeated administration of paroxetine (10 mg/kg/day for 14 days). Each value is the mean ± S.E.M. (n = 6-8 per group). **P < 0.01 as compared with control (saline-treated) group. N.S.: not significant. SPT: sucrose preference test, LMT: locomotion, TST: tail suspension test, FST: forced swimming test.

**Supplementary Figure S3. Effects of ANA-12 on GluA1 levels in the brain regions after withdrawal from repeated METH exposure**

A: Schedule of treatment and Western blot analysis. Saline (10 ml/kg/day for 5 days) or METH (3 mg/kg/day for 5 days) were injected into mice from day 1 to day 5. After 1-week withdrawal, vehicle (10 ml/kg/day, i.p.), ANA-12 (ANA: 0.5 mg/kg/day, i.p.), or 7,8-DHF (DHF: 10 mg/kg/day, i.p.) were administered for 14 days (days 12-25). Sample correction for Western blot analysis was performed at day 28. B: PFC, DG, CA3: There were no differences among the four groups. NAc: Increased GluA1 protein in the NAc of METH-treated mice were significantly attenuated by subsequently repeated administration of ANA-12, but not 7,8-DHF. Each value is the mean ± S.E.M. (n = 6-8 per group). **P < 0.01 as compared with METH + vehicle group. N.S.: not significant.

**Supplementary Figure S4. Lack of effect of bilateral injection of ANA-12 into NAc core on depression-like behavior after withdrawal from repeated METH exposure**

A: Schedule of treatment and behavioral tests. Saline (10 ml/kg/day for 5 days) or METH (3 mg/kg/day for 5 days) were injected into mice from day 1 to day 5. Two days after the final injection, surgery was performed described in the Method section. Vehicle or ANA-12 was injected bilaterally into the NAc core. Behavioral tests were performed 1 hr (LMT), 3 hrs (TST), 5 hrs (FST) after injection of ANA-12. The SPT was performed at days 10. B: LMT: There were no differences among the three groups. TST, FST: Increased immobility time of METH-treated mice was not altered by a single bilateral injection of ANA-12 into the NAc core. SPT: Decreased sucrose preference of METH-treated mice was not altered after a single bilateral injection of ANA-12 into the NAc core. Each value is the mean ± S.E.M. (n = 5-7 per group). *P< 0.05, **P < 0.01 as compared with Control group. N.S.: not significant. LMT: locomotion, TST: tail suspension test, FST: forced swimming test, SPT: sucrose preference test.
